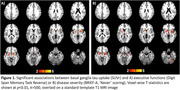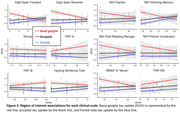# Tau‐PET imaging in Progressive Supranuclear Palsy and relationship with biomarkers and clinical markers

**DOI:** 10.1002/alz.093950

**Published:** 2025-01-09

**Authors:** Roxane Dilcher, Charles B Malpas, Terence J O'Brien, Lucy Vivash

**Affiliations:** ^1^ Monash University, Melbourne, VIC Australia; ^2^ The University of Melbourne, Melbourne Australia; ^3^ Central Clinical School, Monash University, Melbourne, VIC Australia; ^4^ Royal Melbourne Hospital, Parkville, VIC Australia

## Abstract

**Background:**

Identifying biomarkers for primary tau pathologies like Progressive Supranuclear Palsy (PSP) is crucial for diagnosis and treatment development. The novel positron emission tomography (PET) radiotracer 18F‐PI‐2620 shows promise in detecting tau protein in PSP and this study investigates its correlation with clinical markers.

**Methods:**

We conducted a cross‐sectional analysis on 20 patients with clinically diagnosed probable PSP (Richardson’s Syndrome), who underwent T1‐weighted MRI, lumbar puncture, blood testing, and 0‐60 min dynamic 18F‐PI‐2620 PET scanning. Binding potential (BD) and Standardized uptake value ratios (SUVr) were generated for regions of interest. Clinical assessments included the Digit Span Memory task, Hayling, Stroop, Trail Making Test, NIH toolbox subtasks, the BRIEF‐A, and the PSP‐RS. Voxel‐wise and region‐based multiple regression analyses were employed to examine relationship between PET metrics and clinical markers using FSL, SPM, and R software.

**Results:**

Preliminary results revealed a significant negative correlation between basal ganglia tau uptake and the Digit Span Reverse task or the BRIEF‐A (voxel‐wise, SUVr: p<0.01; BD: p<0.05). Region‐based analyses (SUVr) showed basal ganglia tau uptake significantly correlated with impairments in the Digit Span Forward and Reverse task (p<0.01), working memory subtask of the NIH (p<0.05), and the BRIEF‐A (‘Never’‐scores, p<0.05). Significant interaction effects (p<0.05) showed that these associations were predominantly localized in the basal ganglia as compared to frontal or occipital regions. Additionally, basal ganglia atrophy was negatively associated with tau uptake (SUVr) in the basal ganglia and brainstem. Ongoing analysis explores associations with CSF/blood biomarkers (GFAP/NfL/tau).

**Conclusions:**

Our findings highlight the potential of 18F‐PI‐2620 as an in‐vivo tau biomarker in PSP diagnosis, demonstrating significant links between tau uptake in the basal ganglia, brain atrophy, executive dysfunction, and disease severity. These insights could drive the development of new biomarkers, advancing future clinical trial prospects.